# Quantitative evaluation of high intensity signal on MIP images of carotid atherosclerotic plaques from routine TOF-MRA reveals elevated volumes of intraplaque hemorrhage and lipid rich necrotic core

**DOI:** 10.1186/1532-429X-14-81

**Published:** 2012-11-29

**Authors:** Kiyofumi Yamada, Yan Song, Daniel S Hippe, Jie Sun, Li Dong, Dongxiang Xu, Marina S Ferguson, Baocheng Chu, Thomas S Hatsukami, Min Chen, Cheng Zhou, Chun Yuan

**Affiliations:** 1 Department of Radiology, University of Washington, 815 Mercer St, Seattle, WA 98109-4325, USA; 2 Department of Vascular Surgery, University of Washington, Seattle, USA; 3 Department of Radiology, Beijing Hospital, Beijing, China

**Keywords:** Carotid plaques, Intraplaque hemorrhage, Lipid rich necrotic core, Magnetic resonance imaging, Maximum intensity projection

## Abstract

**Background:**

Carotid intraplaque hemorrhage (IPH) and lipid rich necrotic core (LRNC) have been associated with accelerated plaque growth, luminal narrowing, future surface disruption and development of symptomatic events. The aim of this study was to evaluate the quantitative relationships between high intensity signals (HIS) in the plaque on TOF-MRA and IPH or LRNC volumes as measured by multicontrast weighted CMR.

**Methods:**

Seventy six patients with a suspected carotid artery stenosis or carotid plaque by ultrasonography underwent multicontrast carotid CMR. HIS presence and volume were measured from TOF-MRA MIP images while IPH and LRNC volumes were separately measured from multicontrast CMR.

**Results:**

For detecting IPH, HIS on MIP images overall had high specificity (100.0%, 95% CI: 93.0 – 100.0%) but relatively low sensitivity (32%, 95% CI: 20.8 – 47.9%). However, the sensitivity had a significant increasing relationship with underlying IPH volume (p = 0.033) and degree of stenosis (p = 0.022). Mean IPH volume was 2.7 times larger in those with presence of HIS than in those without (142.8 ± 97.7 mm^3^ vs. 53.4 ± 56.3 mm^3^, p = 0.014). Similarly, mean LRNC volume was 3.4 times larger in those with HIS present (379.8 ± 203.4 mm^3^ vs. 111.3 ± 122.7 mm^3^, p = 0.001). There was a strong correlation between the volume of the HIS region and the IPH volume measured from multicontrast CMR (r = 0.96, p < 0.001).

**Conclusion:**

MIP images are easily reformatted from three minute, routine, clinical TOF sequences. High intensity signals in carotid plaque on TOF-MRA MIP images are associated with increased intraplaque hemorrhage and lipid-rich necrotic core volumes. The technique is most sensitive in patients with moderate to severe stenosis.

## Background

Carotid intraplaque hemorrhage (IPH) and lipid rich necrotic core (LRNC) play a critical role in the progression of carotid atherosclerotic disease. The presence of IPH and LRNC in carotid atherosclerotic plaque has been associated with accelerated plaque growth, luminal narrowing and symptomatic events [1,2]. Moreover, recent clinical studies have shown an association between plaques containing LRNC or IPH and an increased number of emboli after carotid artery stenting (CAS) [3-7]. Furthermore, it is reported that the sizes of LRNC and IPH are important factors in the amount of debris produced during CAS, which cause ischemic complications [8-10].

Cardiovascular magnetic resonance (CMR) is noninvasive and has excellent soft tissue contrast. Numerous studies have validated multicontrast CMR assessment of IPH and LRNC against histology in advanced atherosclerotic lesions. These studies have shown it to have high sensitivity and specificity and be capable of volumetric analysis [11,12]. However, these techniques can be time consuming and therefore less suitable for an acute stroke workup or assessment of revascularization procedures. In addition, these techniques are not yet available in the majority of clinical centers. A simple, rapid screening protocol is needed for routine clinical application.

Maximum intensity projection (MIP) images from time-of-flight MR angiography (TOF-MRA) are widely used for screening carotid artery stenosis [13]. This method allows for rapid determination of degree of stenosis and other anatomical findings using rotational views. Moreover, recent work by Yim et al. and Yoshimura et al. have indicated that MIP images from TOF-MRA can also identify IPH as a high intensity signal (HIS) in the plaque [14,15]. This finding raises the possibility of an additional application of MIP images from TOF-MRA for the investigation of carotid artery stenosis. However, these studies did not establish a quantitative link between HIS and LRNC or IPH volume.

As the next step in characterizing HIS and developing it as a quantitative screening method for the evaluation of IPH and plaque burden, we examined the quantitative relationship between HIS in the plaque on TOF-MRA and IPH or LRNC volumes as measured by multicontrast weighted CMR.

## Methods

### Subjects

Eighty three patients with a diagnosis of carotid artery stenosis or the presence of carotid plaque by ultrasonography were recruited from October 2008 to January 2010. Informed consent and IRB approval was obtained from each patient prior to recruitment. The institutional review board approved the study protocol.

### CMR protocol

CMR was performed using a 3.0-T scanner (Achieva; Philips Medical Systems, Best, the Netherlands) and a pair of phased-array carotid coil. One carotid artery was used to center the CMR scan, which was also the side used in image analysis. The symptomatic carotid artery was selected as the index side. Amongst asymptomatic subjects, the more stenotic artery was selected as the index carotid artery. The following six MR contrast weightings were obtained: 3-dimentional (3D) TOF, pre-contrast T1-weighted (T1W) and gadolinium-based contrast enhanced T1-weighted (CE-T1W), proton-density weighted (PDW), T2-weighted (T2W), and 3D magnetization-prepared rapid acquisition gradient-echo (MPRAGE). CE-T1W was acquired six minutes after the injection of gadolinium diethylenetriamine pentacetic acid (Magnevist; Bayer Schering Pharma AG) in a dose of 0.1 mmol/kg body weight at a flow rate of 0.7 ml/sec (Table 1). There were a total of 18 radial projections with an angle of 20 degrees for the MIP images reformatted from 3D TOF MRA. The region of interest was defined to exclude signals from overlying skin and subcutaneous areas.

**Table 1:** Parameters for carotid MR imaging protocols

**Parameter**	T1W^*^	**T2W**	**PDW**	**TOF**	**MPRAGE**
Acquisition mode	2D	2D	2D	3D	3D
Blood suppression technique	QIR	DIR	DIR	-	-
TE (ms)	10	50	9	5	5
TR (ms)	800	4800	4900	20	9
Flip angle (degree)	-	-	-	20	15
FOV	140 x 140	140 x 140	140 x 140	140 x 140	140 x 140
Matrix size	256 x 250	256 x 250	256 x 250	256 x 250	256 x 250
No. of sections	20	20	20	24	24
Section thicknes (mm)	2	2	2	2	2
Coverage (mm)	40	40	40	48	48
Scan time (min)	6	3	3	3	3

### MR image evaluation

#### Multicontrast CMR

Two reviewers, blinded to clinical information and TOF-MRA MIP results, matched and registered multicontrast MR images using the carotid bifurcation as a landmark. Image quality was rated on a four-point scale determined by the overall signal-to-noise ratio (SNR) and the clarity of vessel wall boundaries (1 = poor, 4 = excellent) [16]. Images with a rating < 2 were excluded from the study. The lumen, outer wall, and tissue components were identified and delineated according to consensus opinion using the computer-aided system for cardiovascular disease evaluation (CASCADE), a specialized software suite for plaque analysis [17]. Areas of intraplaque hemorrhage (IPH) and lipid-rich necrotic core (LRNC) were measured using previously published criteria that have been validated by histology [12,18,19]. IPH and LRNC volumes were calculated by summing the products of cross-sectional areas and the corresponding slice thicknesses using CASCADE.

#### High intensity signal on TOF-MRA MIP images

**Figure 1:**
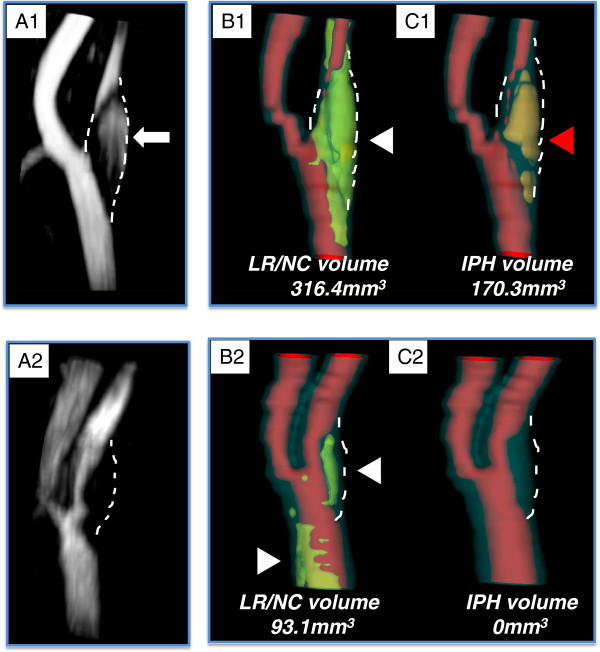
**Representative images of high intensity signal (HIS) positive plaque (A1) and HIS-negative plaque (A2). **The HIS-positive plaque shows a hyperintense region in the vessel wall without connection to the lumen (white arrow). The HIS-negative plaque shows no such hyperintense region in the vessel wall but did contribute to luminal stenosis. Lipid-rich necrotic core (LRNC) (**B1 & 2**; white arrowheads) and intraplaque hemorrhage (IPH) (**C1 & 2**; red arrowheads) are displayed using three dimensionally reconstructed images. The volume of LRNC and IPH were calculated by summing the products of cross-sectional areas and corresponding slice thicknesses.

A high intensity signal (HIS) located in the plaque but having no connection to the lumen in all nine projections was defined as HIS positive (Figure 1). Two reviewers (KY and JS) independently read the TOF-MRA MIP images of the carotid bifurcation and the proximal internal carotid artery while blinded to the clinical information and multicontrast CMR results and recorded whether each was HIS positive or negative. One month after completing the first reading of the MIP images, one reviewer (KY) re-read the cases blinded to the previous results in order to assess inter- and intra-observer agreement. Lastly, the two reviewers discussed their separate readings and arrived at a consensus opinion on the presence/absence of HIS for all arteries. The volume of the HIS region was calculated from cross-sectional areas using CASCADE.

#### Degree of carotid stenosis

The degree of stenosis for each artery was measured following guidelines established by the North American Symptomatic Carotid Endarterectomy Trial using MIP images reformatted from TOF-MRA [20]. The arteries were subsequently divided into three groups according to their level of stenosis: ≤ 15%, 16 – 49%, and ≥ 50%.

### Statistical analysis

Inter- and intra-observer agreement were summarized using Cohen’s kappa and 95% confidence intervals. Continuous variables were summarized as the mean ± SD. The Mann–Whitney *U* test was used to compare right-skewed continuous variables between groups. The classification performance of HIS was summarized with sensitivity and specificity. Sensitivity of HIS was also computed for individual IPH volume and stenosis subgroups. Linear regression was used assess the association between IPH and LRNC volumes and between IPH and HIS volumes. The chi-squared test for trend or Spearman’s rank correlation were used to assess trends between ordered categorical variables and binary or continuous outcomes, respectively. A value of p < 0.05 denoted statistical significance. All statistical analyses were performed using R 2.14.1 (R development Core Team (2011). R Foundation for Statistical Computing, Vienna, Austria).

## Results

Of the 83 patients scanned, 7 were excluded during image review due to insufficient image quality or scan coverage of the index artery. Amongst the remaining 76 patients available for analysis, 62 (81.6%) were men with an age between 38 and 86 years (mean: 66). Eight (11%) arteries showed a high intensity signal (HIS) on MIP images (HIS-positive plaque). Sixty eight arteries were HIS-negative on MIP images (Figure 1). There was good inter-observer agreement (kappa = 0.78 [0.53 – 1.00]) and good intra-observer agreement (kappa = 0.84 [0.63 – 1.00]).

### Association between HIS and the presence and size of IPH and LRNC

**Figure 2:**
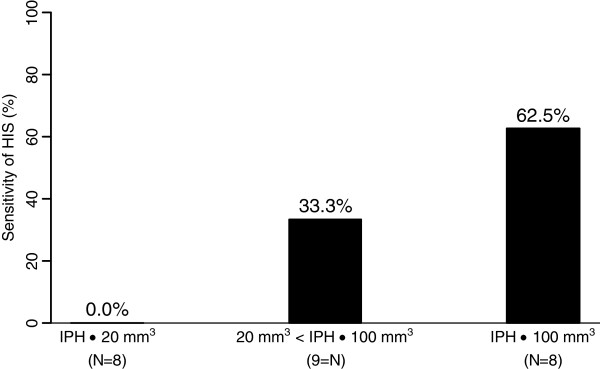
**Sensitivity of the high intensity signal (HIS) on TOF-MRA MIP images to detect intraplaque hemorrhage (IPH), grouped by underlying IPH volumes. **Subgroups were chosen to achieve approximately equal sample sizes. There was a significant increasing trend between sensitivity of HIS and IPH volume (p=0.007).

There were 25 (33%) and 73 (96%) subjects with IPH and LRNC by multicontrast CMR, respectively. The sensitivity and specificity of HIS for IPH were 32% (95% CI: 20.8 – 47.9%) and 100% (95% CI: 93.0 – 100.0%), respectively. In particular, all HIS positive plaques contained IPH. The sensitivity and specificity of HIS for LRNC were 11% (95% CI: 4.9 – 20.5%) and 100% (95% CI: 29.2 – 100.0%). The sensitivity for IPH improved as IPH volume increased (p = 0.007) (Figure 2).

**Figure 3:**
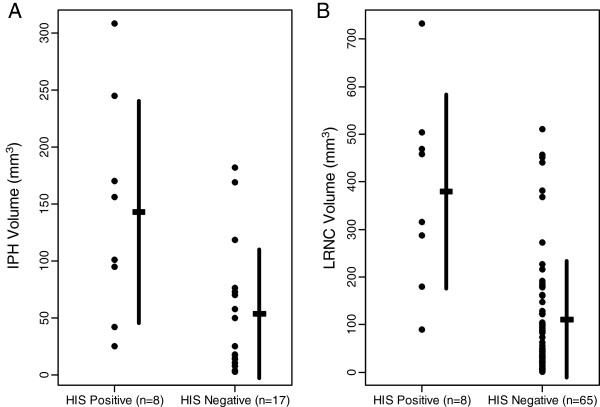
**(A) Intraplaque hemorrhage (IPH) volume assessed by multicontrast weighted CMR comparing high intensity signal (HIS) positive and HIS-negative plaques by TOF-MRA MIP images in the patients with carotid IPH. **IPH volume is significantly larger in the HIS-positive plaque group than the HIS-negative group. (**B**) Lipid-rich necrotic core (LRNC) volume assessed by multicontrast CMR comparing HIS positive with HIS-negative plaques by MIP in the patients with carotid LRNC. LRNC volume was also significantly larger in the HIS-positive plaque group than in the HIS-negative group.

**Figure 4:**
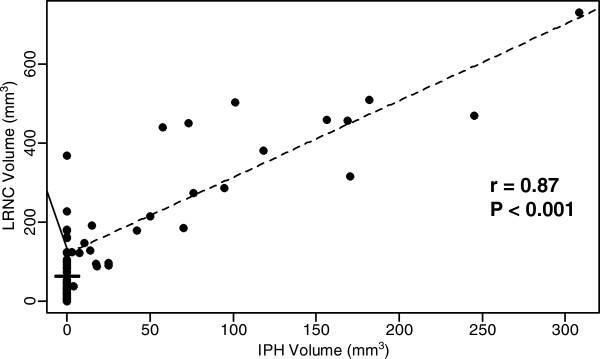
**The relationship between intraplaque hemorrhage (IPH) and lipid rich necrotic core (LRNC) volumes. **There was a strong positive correlation between IPH volume and LRNC volume.

Mean IPH volume was 2.7 times larger in the HIS-positive group than in the HIS-negative group among the 25 subjects with IPH present (142.8 ± 97.7 mm^3^ vs. 53.4 ± 56.3 mm^3^, p = 0.014) (Figure 3). Similarly, mean LRNC volume was 3.4 times larger in the HIS-positive group than in the HIS-negative group among the 73 subjects with LRNC present (379.8 ± 203.4 mm^3^ vs. 111.3 ± 122.7 mm^3^, p = 0.001) (Figure 3). There was a significant positive correlation between IPH volume and LRNC volume (r = 0.87, p < 0.001) (Figure 4).

**Figure 5:**
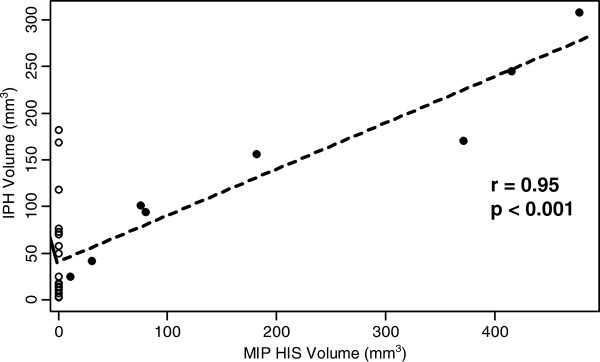
**The relationship between volume of the high intensity signal (HIS) on TOF-MRA MIP images and intraplaque hemorrhage (IPH) volume as measured by multicontrast CMR in HIS-positive plaques (solid circles). **The open circles show IPH volumes in HIS-negative plaques with IPH for visual reference (see Figure 3). There was a strong positive correlation between MIP and IPH volumes, though the MIP volumes tended to be larger.

In the eight arteries with HIS positive plaques, the volume of the HIS region was compared with the IPH volume measured from multicontrast CMR (Figure 5). There was a strong correlation between the two measurements (r = 0.96, p < 0.001), though the MIP volumes tended to be larger than the corresponding IPH volumes.

### Comparison with degree of stenosis

Arteries were grouped by stenosis into three categories: 0-15%, 16-49% and 50-99%. As shown in Table 2, there was a significant increasing trend between stenosis categories and IPH presence. In those arteries with IPH, there was also a significant increasing trend between stenosis and IPH volume. Of note, IPH was found in 13.5% of arteries with 0-15% stenosis. Furthermore, the sensitivity of HIS to detect IPH increased with greater degrees of stenosis (p = 0.022). The observed specificity of HIS was 100% in all three groups.

**Table 2:** Relationships between carotid stenosis, intraplaque hemorrhage (IPH) presence and size, and sensitivity/specificity of the high intensity signal (HIS) on TOF-MRA MIP images for IPH classification

**Stenosis**	**N (%)**	**% with IPH**	**Mean IPH**	**Sensitivity (95% CI)**	**Specificity**
			**Volume (mm**^**3**^)*		**(95% CI)**
**0 – 15%**	37 (48.7)	13.5	45.7	0.0 (0.0 – 52.2)	100.0 (89.1 – 100.0)
**16 – 49%**	21 (27.6)	42.9	52.2	22.2 (2.8 – 60.0)	100.0 (73.5 – 100.0)
**50 – 99%**	18 (23.7)	61.1	122.9	54.5 (23.3 – 83.3)	100.0 (59.0 – 100.0)
**p for trend**	-	< 0.001^†^	0.030^‡^	0.022^†^	1.000^†^

^*^In those with IPH present. ^†^Chi-squared test for trend. ^‡^Spearman’s rank correlation. *CI* = confidence interval.

## Discussion

This study demonstrated that IPH and LRNC volumes were significantly larger in HIS positive plaques than in HIS negative plaques (Figure 3). This study also demonstrated a strong correlation between MIP and cross-sectional multicontrast weighted images for the evaluation of IPH volume.

### IPH detection using HIS

The sensitivity for presence of IPH was relatively low for the sample as a whole but significantly increased as the volume of IPH increased (Figure 2). Furthermore, the specificity was high as HIS was not observed in any arteries without IPH.

Since the prevalence of IPH and its volume both tended to increase with level of stenosis, a significant positive association was also seen between stenosis and sensitivity. Even in the lowest category of stenosis considered, 0-15%, 13.5% of the arteries had IPH, none of which were detected using TOF-MRA MIP images. These findings regarding HIS have not been reported before because previous studies evaluating TOF-MRA MIP images focused on arteries with moderate to severe stenosis only [14,15]. As IPH has been found to be a high-risk indicator in multiple populations, a more sensitive method may be needed when evaluating those with low-grade stenosis. It should be noted that others have also pointed out that high risk features can be seen in those with minimal angiographic evidence of disease [21,22] and that strokes often occur in those with zero to moderate stenosis [23,24].

While HIS appears to have limited sensitivity in those with low-grade stenosis, it was found to be more reliable in those with moderate to severe stenosis and to have strong correlations with IPH and LRNC size when present. As such patients may be candidates for endovascular intervention and it has been demonstrated that large amounts of LRNC and IPH are associated with ischemic complications during and after CAS. HIS on TOF-MRA MIP images has potential for effective preoperative screening and evaluation [8-10,25]. Accordingly, a recent study demonstrated that performing CEA in those with HIS positive plaques rather than CAS resulted in fewer periprocedural strokes [15].

### Advantages of TOF-MRA MIP image screening for IPH

In this study, the multicontrast sequences used to evaluate IPH included MPRAGE. Ota et al reported that MPRAGE, as compared with the TOF sequence, had higher sensitivity in the detection of IPH at 3 T [19]. However, MPRAGE does not appear to be suitable as a singular screening method for evaluating carotid stenosis primarily because of insufficient blood suppression which limits its use for evaluating the lumen [26]. Therefore, in light of the evidence of a strong quantitative relationship with IPH and LRNC in those with moderate to severe stenosis, the three minute acquisition time and wide availability across clinical centers, we consider TOF-MRA MIP images a suitable technique for the evaluation of carotid disease in a select but critical population of patients.

### Study limitations

The present study had a number of limitations. First was the lack of a histological gold standard. In this study there were no patients who received CEA, therefore we did not have histology available. However, the multicontrast CMR protocol used has previously been shown to have good correspondence to histology [11,12,19] and the presence of HIS in the plaque on MIP images from TOF MRA has also been validated by histology [14,15]. Second was the modest number of patients and the low number of HIS positive plaques. While strong relationships were evident even at this sample size, a larger study would improve precision and be more definitive. Third, images were acquired on a 3 T CMR scanner using dedicated carotid coils. Further studies using conventional head/neck or neurovascular coils on 1.5 T and 3 T CMR scanner are needed to determine whether these study findings can be generalized to the clinical setting. One strength of this study relative to others is the broad range of stenoses present which enabled a more complete assessment of HIS on TOF-MRA.

## Conclusion

High intensity signals (HIS) in carotid plaque on TOF-MRA MIP images are associated with an increased size of intraplaque hemorrhage (IPH) and lipid rich necrotic core (LRNC), particularly in those with moderate to severe stenosis. MIP images are easily reformatted from three minute, routine, clinical TOF sequences. Since IPH was frequently detected in arteries with low grade stenosis—where TOF-MRA MIP images were found to have low sensitivity—methods with higher sensitivity for IPH while preserving the desirable capability of visualizing the lumen in three dimensions are needed to better evaluate this subpopulation.

## Competing interests

The authors declare that they have no competing interests.

## Authors’ contributions

KY was in charge of the MR image review and interpretation of data, preparation of the manuscript. YS was in charge of the MR image collection and interpretation of data. DSH contributed to the data analysis, interpretation and presentation of results and revision of the manuscript. JS and LD reviewed and peer-reviewed MR images. DX assisted in data interpretation. MSF assisted in data interpretation and manuscript revision. BC assisted in development of the study design and interpretation of data. He also significantly revised the manuscript. TSH assisted in data interpretation and was key to revising the critical content of the manuscript. MC and CZ were involved with revision of the manuscript. CY was involved with study design, data interpretation and data analysis. All authors have read and approved submission of this manuscript. The material in the manuscript has not been previously published and is not being considered for publication elsewhere in whole or in part in any language.
